# Supplementary material for: Deletion of wfs1 Impairs Oligodendrocyte Precursor Cells Dorsal Distribution and Myelination Through the wfs1-hmgcs1 Axis in Zebrafish
Source: Biology (Basel). 2026 Aug 16;15(16):1402. doi: 10.3390/biology15161402 (PMC13510151; doi:10.3390/biology15161402)
Supplement: Supplementary file 1 [file biology-15-01402-s001.zip › File S1-Transcriptome sequencing data and differentially expressed genes (DEGs) in wfs1-- zebrafish at 5 dpf/2.KEGG/wfs1KOvsWT/UP/src/dre04218.html]

dre04218


- dre:557551

- Up regulated genes

557551\_1(1.43805955630111)

- dre:100000252

- Up regulated genes

100000252\_3(1.94434154854557) 100000252(1.09163460881879)

- dre:100330617

- Up regulated genes

100330617\_1(1.54741913113035)

- dre:562552

- Up regulated genes

562552(1.50682290794657)

- dre:431763

- Up regulated genes

431763(1.93865999132)

- dre:393176

- Up regulated genes

393176(7.71939682866725)

- dre:767751

- Up regulated genes

767751\_1(3.05541031760882)

- dre:333941

- Up regulated genes

333941\_1(2.47762653802975)

- dre:192322

- Up regulated genes

336121(1.42904963424916)

- dre:565241

- Up regulated genes

565241(1.32977867526604)

- dre:559723

- Up regulated genes

559723(3.63719210583924)

- dre:559723

- Up regulated genes

559723(3.63719210583924)

- dre:100000252

- Up regulated genes

100000252\_3(1.94434154854557) 100000252(1.09163460881879)

- dre:393186

- Up regulated genes

393186\_1(3.79579966743607)

Close
